# Arousal Contributions to Resting-State fMRI Connectivity and Dynamics

**DOI:** 10.3389/fnins.2019.01190

**Published:** 2019-11-05

**Authors:** Yameng Gu, Feng Han, Xiao Liu

**Affiliations:** ^1^Department of Biomedical Engineering, The Pennsylvania State University, University Park, PA, United States; ^2^Institute for CyberScience, The Pennsylvania State University, University Park, PA, United States

**Keywords:** resting-state fMRI, brain dynamics, arousal, global signal, connectivity

## Abstract

Resting-state functional magnetic resonance imaging (rsfMRI) is being widely used for charting brain connectivity and dynamics in healthy and diseased brains. However, the resting state paradigm allows an unconstrained fluctuation of brain arousal, which may have profound effects on resting-state fMRI signals and associated connectivity/dynamic metrics. Here, we review current understandings of the relationship between resting-state fMRI and brain arousal, in particular the effect of a recently discovered event of arousal modulation on resting-state fMRI. We further discuss potential implications of arousal-related fMRI modulation with a focus on its potential role in mediating spurious correlations between resting-state connectivity/dynamics with physiology and behavior. Multiple hypotheses are formulated based on existing evidence and remain to be tested by future studies.

## Introduction

The advent of resting-state functional magnetic resonance imaging (fMRI) (Biswal et al., 1995; Fox and Raichle, 2007) has revolutionized our understanding of large-scale brain networks, including their intrinsic organization (Fox et al., 2005), developmental and aging profiles (Fair et al., 2007; Stevens et al., 2008), state-dependent re-organization (Horovitz et al., 2009; Barttfeld et al., 2014), genetic basis (Wiggins et al., 2012), and most importantly their modulations in various brain diseases (Zhang and Raichle, 2010). The majority of studies in this research field has been focused on inferring functional brain connectivity with fMRI correlations. The majority of these studies estimated functional connectivity based on an entire session of typical 5–10 min, which implicitly assumes stationary relationships between different brain regions and ignores temporal brain dynamics at finer time scales of seconds. Recently, the non-stationary nature of resting-state fMRI (rsfMRI) became a hot topic of this research field (Chang and Glover, 2010) and a set of methods/metrics has been proposed to extract and quantify time-varying information in rsfMRI data, which is expected to provide information supplementary to those stationary analyses (Hutchison et al., 2013; Preti et al., 2017).

The approaches for quantifying rsfMRI dynamics can be divided into multiple categories. The most straightforward class is sliding window approaches, which quantify rsfMRI connectivity within short time windows of 1–2 min and then examine its temporal variability accordingly. The connectivity metrics could be either conventional correlation/coherence (Chang and Glover, 2010; Allen et al., 2012) or more sophisticated metrics, such as network parameters from graph-theory based quantifications (Braun et al., 2015; Shine et al., 2016). A group of single-volume resolved methods has also been proposed to quantify rsfMRI dynamics. These methods treat fMRI volumes at single time points as basic units of analyses and try to identify repeated patterns of brain co-activations using different algorithms, including the temporal functional mode (TFM) extracted with temporal independent component analysis (ICA) (Smith et al., 2012), the co-activation patterns (CAP) identified by clustering (Liu and Duyn, 2013b; Liu et al., 2013), and the brain states defined using hidden Markov model (HMM) (Chen et al., 2016; Vidaurre et al., 2017). Subsequent quantification can then be applied to quantify temporal dynamics, such as occurrence rate and transitioning probabilities, of these single-volume fMRI events. The third category of dynamic approaches expands the second type by focusing on spatiotemporal structures in rsfMRI signals. Different algorithms were employed to derive quasi-periodic patterns (QPP) (Thompson et al., 2014) and lag threads (Mitra et al., 2015a) from rsfMRI data that may represent propagating activities of the brain.

**FIGURE 1:**
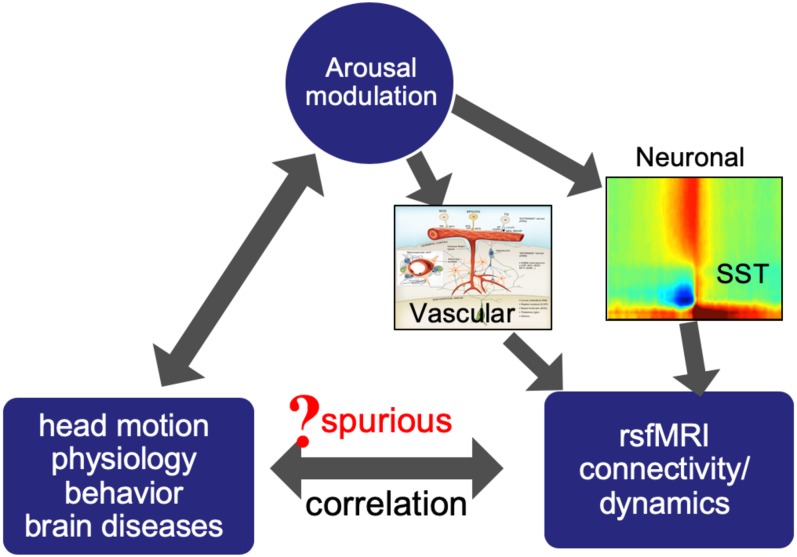
Arousal modulations may introduce spurious correlations between rsfMRI connectivity/dynamics and other measures by modulating both.

These dynamic approaches have been applied to rsfMRI data to quantify brain dynamics and investigate its associations with behavior (Vidaurre et al., 2017) and modulations under pathological conditions (Mitra et al., 2015b). A very consistent observation across studies and species is the sensitivity of fMRI dynamics to brain states showing distinct arousal levels (Barttfeld et al., 2014; Tagliazucchi and Laufs, 2014; Liang et al., 2015; Mitra et al., 2015c; Ma et al., 2016; Laumann et al., 2017). The brain arousal is conventionally defined as a transient intrusion of being awake into sleep stages or an abrupt temporary increase of the vigilance level (Atlas Task Force, 1992; Halász et al., 2004), and the sleep and anesthesia conditions are known to show distinct arousal levels. In particular, the application of a wake-sleep classifier trained based on dynamic functional connectivity of a small EEG-fMRI data set to a large cohort of 1,147 rsfMRI datasets has found that 30% subjects actually fell asleep within 3 min into resting-state scanning (Tagliazucchi and Laufs, 2014). These findings not only suggest an important role of arousal in generating/modulating rsfMRI signals and thus connectivity/dynamics measures derived from it, but also imply its prevalent influence on human rsfMRI studies. Consistent with these observations, a characteristic neurophysiological event signifying a transient arousal modulation was identified recently and shown to have profound effect on concurrently acquired fMRI signals (Liu et al., 2015, 2018), which give us an opportunity of further looking into the relationship between the arousal and rsfMRI signals. In this perspective, we will first review the relationship between the brain arousal and global rsfMRI signal and a recently discovered neurophysiological event that may underlie this relationship. We will then discuss potential implications of these findings on different aspects of rsfMRI research (Figure 1), including the motion-rsfMRI, physiology-rsfMRI, and disease-rsfMRI relationships in different sections.

## A Neurophysiological Event Underlying the Global RSFMRI Signal

The global rsfMRI signal averaged over the entire brain and spatially non-specific fMRI correlations it induces have been found highly sensitive to brain arousal state (Matsuda et al., 2002; Schölvinck et al., 2010). The transition into the light sleep is characterized by a large increase in this global rsfMRI signal (Fukunaga et al., 2006; Horovitz et al., 2008; Larson-Prior et al., 2009), and a similar but smaller change was also observed from an alert eyes-open condition to a sleep-conducive eyes-closed condition (Wong et al., 2013; Xu et al., 2014; Wei et al., 2018; Agcaoglu et al., 2019). Caffeine can effectively reduce the global rsfMRI signal and corresponding EEG vigilance index (Wong et al., 2013), whereas several hypnotic drugs (Kiviniemi et al., 2005; Saper et al., 2005; Greicius et al., 2008; Licata et al., 2013) and sleep deprivation (Yeo et al., 2015) had the opposite effects. It is also worth noting that multiple studies also showed the connectivity changes of the default mode network (DMN) under various states of consciousness (Yan et al., 2009; Vanhaudenhuyse et al., 2010; Heine et al., 2012; Demertzi et al., 2015). These studies provided convincing evidence for a close relationship between the arousal and rsfMRI, particularly its global component, but the neural basis underlying this relationship had remained unknown until very recently.

The first clue for the neural basis of the global rsfMRI signal came from the study of rsfMRI dynamics. It has been suggested that the rsfMRI connectivity and its dynamics are actually caused by brain co-activations at different time points that can be captured by CAPs (Liu and Duyn, 2013b; Liu et al., 2013; Matsui et al., 2018). Applying the CAP decomposition to rsfMRI data with and without removing the global signal showed distinct effects on two types of CAPs. Whereas the global signal regression (GSR) procedure has very limited effect on higher-order CAPs, e.g., those related to the DMN, it dramatically changes sensory CAPs involving the sensorimotor and visual cortices (Liu and Duyn, 2013a; Liu et al., 2013; Nalci et al., 2017). The finding not only confirmed that the global signal is largely driven by global rsfMRI co-activations occurring only at a proportion of time points, but also implied the neuronal origin of this fMRI co-activation since it occurs preferentially with sensory networks rather than randomly. These results are consistent with another series of research work on the temporal heterogeneity of the global rsfMRI signal (He and Liu, 2012; Nalci et al., 2017). With these findings, the search for the neurophysiological correlate of the global rsfMRI signal was shifted to the event type of neuronal processes.

An electrophysiological event signifying a transient arousal modulation was recently discovered and suggested to underlie the global rsfMRI signal (Liu et al., 2015, 2018). This sequential spectral transition (SST) event was first observed in the global signal of a large-scale electrocorticography (ECoG) recording from monkeys, showing as a stereotypic time-frequency pattern of sequential power changes at three distinct frequency bands: a large, sudden reduction in the middle-frequency (9–21 Hz) power is followed by an increased broad-band high-frequency gamma power (>42 Hz) and then a burst of low-frequency delta waves (1–4 Hz). The SST lasts 10–20 s and shows similar state-dependency as the global rsfMRI signal (Liu et al., 2015). Concurrent fMRI-electrophysiology recordings from another group of monkeys confirmed that the SST induces widespread fMRI increases, i.e., the global rsfMRI co-activation, shown as a large peak in the global signal (Liu et al., 2018). In addition to such one-to-one correspondence between the SST and global rsfMRI peak, their relationship was further confirmed from the other two aspects. First, the global rsfMRI co-activation demonstrated a much larger amplitude in sensory regions, i.e., the sensorimotor, auditory, and visual cortices, and this sensory-dominant pattern is consistent with the spatial pattern of the high-frequency gamma (40–90 Hz) power increase at the SST event. Second, the global co-activations are associated with very specific de-activations at subcortical arousal-promoting areas, i.e., the Nucleus Basalis (NB) at the basal forebrain and the midline thalamus in the non-specific arousal pathway, in accordance with a transient arousal drop suggested by the middle-to-low frequency spectral transition at SST events (Liu et al., 2018). Consistent with this finding, the inactivation of the NB in one brain hemisphere of monkeys resulted in a significant reduction of the global rsfMRI signal in the ipsilateral side (Turchi et al., 2018). Overall, the finding of the SST event and its relationship with fMRI signals provide a neurophysiological understanding of the relationship between the brain arousal states and rsfMRI connectivity/dynamics.

Arousal-related fMRI changes can have potential implications in many aspects of rsfMRI research. The sensory-dominant pattern of SST-induced fMRI changes is expected to introduce very systematic changes in rsfMRI correlations, which could be easily misinterpreted as meaningful modulations of functional brain connectivity. The transient nature of the SST (10–20 s) will also have profound effects on rsfMRI dynamics at the time scale of interest to most of dynamic rsfMRI studies. More importantly, the perils of the arousal-related fMRI component can go beyond its direct effects on rsfMRI connectivity/dynamics by potentially introducing their spurious correlations with other subject measures of physiology, behavior, and pathology. Arousal state is known to have profound effects on physiology (Trinder et al., 2001) and also able to affect behavioral performance (Teigen, 1994) or even head motions (Van Den Berg, 2006). Many brain diseases, including Alzheimer’s disease (Musiek et al., 2015), Parkinson’s disease (Breen et al., 2014), and autism spectrum disorders (ASD) (Cohen et al., 2014), are known to concur with disrupted sleep and circadian rhythms (Wulff et al., 2010), and many medications for these diseases are also known to affect brain arousal state. Together with the profound effects of arousal modulation on rsfMRI signals, these may lead to spurious relationships between rsfMRI connectivity/dynamics and various physiological and behavioral measurements (Figure 1). The remaining part of this perspective will have detailed discussions regarding the role of arousal modulations in mediating the relationship of rsfMRI with different types of subject measurements.

## The Potential Role of Arousal Modulations in Motion-RSFMRI Relationship

The correlation has been found between the rsfMRI connectivity and head motions in both intra- and inter-subject analyses (Power et al., 2012; van Dijk et al., 2012; Yan et al., 2013). Specifically, more head motions are associated with increased local but reduced long-range rsfMRI connectivity. This motion-connectivity association has been interpreted as a causal relationship with assuming that the head motion affects fMRI signals and thus their correlations. However, there are observations inconsistent with this interpretation. First, the motion-associated rsfMRI signal/correlation change persists or even reaches its peaks 10 s after motion ceases (Power et al., 2014; Byrge and Kennedy, 2018). This temporal feature cannot be caused by the spin-history artifact, which should have a much short delay to the motion according to simulation and also monotonically decay over time (Yancey et al., 2011). Instead, this time delay is in a similar time scale as the typical hemodynamic delay. Secondly, the associated rsfMRI connectivity changes showed systematic spatial patterns that are unexpected from relatively random head motions. Thirdly, the same amount of head motions causes significant rsfMRI connectivity changes across subjects but not between different sessions of the same subjects (Zeng et al., 2014). For these reasons, the motion-connectivity relationship may not be causal, but actually mediated by a third factor. We propose that the arousal modulation could be a candidate that mediates this relationship for the following reasons. First, a widely used motion index, differentiated signal variance (DVARS), detects large fMRI changes, including large global signal peaks that have been linked to the SST event of arousal modulation. Secondly, the motion-fMRI correlations also show a sensory-dominant pattern similar to that of the global co-activations and SST gamma power (Yan et al., 2013). Thirdly, sleepiness has been shown to be associated with more head motions (Van Den Berg, 2006). Transient sleep structures, such as microsleep and/or microarousal, and associated physiological modulations might be direct causes of head motions. Indeed, a transient respiratory modulation was found to concur with head motions detected by fMRI changes (Byrge and Kennedy, 2018). For all these reasons, we hypothesize that transient arousal modulations induce spurious correlations between the head motion and rsfMRI connectivity, which account for a significant proportion of the observed motion-rsfMRI relationships. The key to validating this hypothesis is to differentiate the head motions of arousal relevance from those caused by discomfort, general fidgetiness, and other factors, as well as their effects on rsfMRI signals. It is worth noting that the framewise displacement (FD), another widely used motion index calculated directly from image alignment parameters (Yoo et al., 2005), might better serve this purpose, compared with DVARS, with less contamination from the arousal-related global signals.

## The Potential Role of Arousal Modulations in Physiology-RSFMRI Relationship

Physiological signals, including respiratory volume and cardiac pulse rate, were also shown to have strong correlations with rsfMRI signals (Birn et al., 2006; Shmueli et al., 2007; Chang et al., 2016b; Özbay et al., 2018), which has been regarded as evidence of non-neuronal contributions to rsfMRI signal fluctuation. A recent study combing fMRI, physiology, and electroencephalogram (EEG) provided further insight into this physiology-rsfMRI relationship (Yuan et al., 2013). It first confirmed the correlation between the physiology and rsfMRI but further suggested that they both are also correlated with the alpha-band EEG power, which is an indicator of brain vigilance and also shows a large modulation at the SST. Moreover, this study further showed the physiology-rsfMRI correlation is dependent on brain states and significant only during the sleep-conducive eyes-closed condition but not under an alert eyes-open condition. It is worth noting that the correlations between rsfMRI and physiological signals also appear to be much stronger in the sensory regions than the rest of the brain (Birn, 2006; Shmueli et al., 2007; Yuan et al., 2013; Özbay et al., 2018). All these findings strongly suggest an involvement of the arousal in this physiology-rsfMRI relationship. We thus hypothesize that the physiology-rsfMRI relationship is partly caused by their co-modulations at transient arousal events, such as the SST. Given the potential involvement of physiology, we want to also emphasize that the arousal modulation may cause fMRI changes via two different routes. It can modulate neural activities across the cortex via the ascending arousal pathways, and thus fMRI signal changes through local neuro-vascular coupling. The SST event and associated global rsfMRI co-activation are likely evidence for this type of arousal-fMRI connections. In addition, the brain stem arousal centers are also able to directly modulate vascular tone, for example, through sympathetic innervations of the arteries in the brain pial surface (Hamel, 2005; Özbay et al., 2018), and thus cause global rsfMRI changes of vascular origin. Large white-matter fMRI changes associated with cardiac signal changes likely originate from this type of vascular modulations (Özbay et al., 2018). Differentiating the contributions from these two mechanisms remains a challenge for future research.

## The Potential Role of Arousal Modulations in Disease-RSFMRI Relationships

Resting-state fMRI connectivity and dynamics have also been extensively studied under pathological conditions, and significant modulations were reported in a wide range of neurological disorder and psychiatric diseases, including the Alzheimer’s disease, ASD, and major depression (Zhang and Raichle, 2010). Given that these brain diseases are often associated with disrupted sleep and circadian rhythms (Wulff et al., 2010), it is reasonable to suspect that the arousal difference may, at least partly, account for rsfMRI connectivity modulations observed in certain brain diseases. A survey of existing literatures indeed found the evidence for the modulation of the global rsfMRI signal under certain pathological conditions. A simple example is schizophrenia. Whereas an early study had suggested that schizophrenia patients showed hyperconnectivity in the default network compared with their first-degree relatives and healthy controls (Whitfield-Gabrieli et al., 2009), it was found later that these changes may actually arise from an enhanced global signal in schizophrenia (Yang et al., 2014). A computational model was also employed to demonstrate that increased neuronal coupling can indeed enhance the global signal (Yang et al., 2014). However, based on the evidence we reviewed so far regarding the relationship between the global rsfMRI signal and arousal, we argue that the distinct arousal state could be an alternative explanation for the global rsfMRI signal seen in the schizophrenia patients.

**FIGURE 2:**
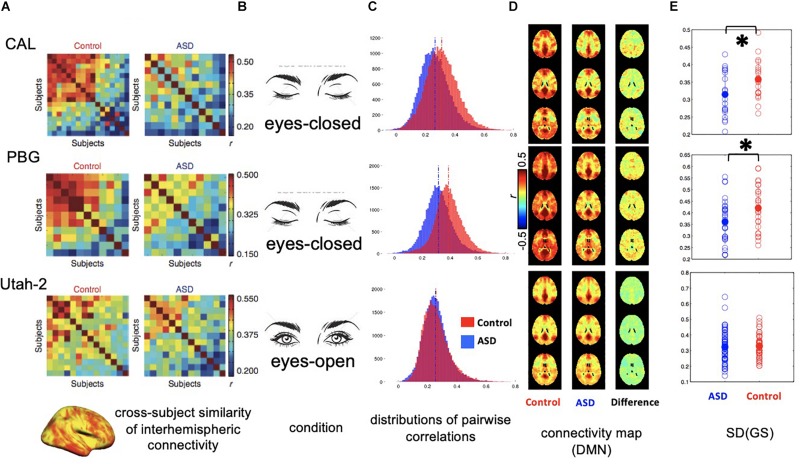
Distinct global signal in ASD patients may have implicit impact on interhemispheric rsfMRI connectivity. **(A)** Correlation matrices showing the inter-subject similarity of interhemispheric rsfMRI connectivity pattern for three datasets collected at different sites. The data from the first two sites, i.e., CAL and PBG show a big difference between the ASD and control groups with controls showing much higher cross-subject similarities. **(B)** Conditions for rsfMRI experiments. The first two sites collected rsfMRI data under sleep-conducive eyes-closed condition. **(C)** Histograms showing the distribution of all pairwise rsfMRI connectivity. In the first two datasets, the control groups show overall stronger rsfMRI connectivity compared with the ASD groups. However, this difference was not observed in the third dataset. **(D)** RsfMRI connectivity maps with respect to a seed region at the posterior cingulate cortex (PCC) showing the DMN network. In the first two datasets, the control groups show larger spatially non-specific correlations compared with the ASD groups, presumably due to a larger global signal. **(E)** Standard deviation of the global rsfMRI signal. The control groups of the first two datasets are characterized by significantly larger global signal than the ASD groups, whereas the global signal is smaller and not different in the two groups for the third dataset. ^∗^0.01 < *p* < 0.05.

The global signal could affect rsfMRI findings in a rather implicit way. Using rsfMRI data from the Autism Brain Imaging Data Exchange (ABIDE) initiative, a previous study has shown that interhemispheric rsfMRI connectivity shows a much larger inter-subject variability in high-functioning ASD adults than matched healthy controls, and this finding has been interpreted as idiosyncratic distortions of ASD brains (Hahamy et al., 2015). The interhemispheric rsfMRI connectivity often shows a sensory-dominant pattern due to strong bilateral correlations in sensory regions. Given the sensory-dominant pattern of the global rsfMRI co-activations of arousal relevance (Liu et al., 2018), the presence of a strong global signal is also expected to enhance this pattern and thus its cross-subject similarity. We therefore hypothesize that the difference in the global rsfMRI signal is responsible for the interhemispheric connectivity difference between the ASD and control groups. To test this hypothesis, we examined ABIDE datasets from three different sites (Figure 2). For two datasets (CAL: 19 ASD and 19 controls; PBG: 30 ASD and 27 controls) that previously showed a large contrast between the ASD and control groups, rsfMRI data were actually acquired under the sleep-conducive eyes-closed condition (Figures 2A–C, top and middle). Moreover, the control group shows a much stronger global signal and rsfMRI connectivity than the ASD group (*p* = 0.036 for CAL and *p* = 0.022 for PBG, 2-sample *t*-test; Figures 2D,E, top and middle). In contrast, the Utah dataset (58 ASD versus 43 controls) not showing much group difference in the previous study was acquired under a more alert eyes-open condition and their global signals are not significantly different (*p* = 0.735; Figures 2A–C, bottom). Correspondingly, the global signal is low in both ASD and control groups for this dataset (Figures 2D,E, bottom). These preliminary results clearly suggest that the ASD groups are characterized by the global rsfMRI signal distinct from healthy controls, especially under the sleep-conducive eyes-closed condition, which might be attributed to their abnormal sleep patterns (Devnani and Hegde, 2015).

Arousal might also mediate the correlation between rsfMRI connectivity/dynamics and certain behavioral measures within the healthy population in a similar way. Even though the arousal itself describes a brain state that varies over time, the ability of regulating arousal could be an individual trait that is stable within but varies across individuals. If the healthy population contains subgroups that not only have distinct ability of regulating arousal but also differ significantly in certain cognitive functions, rsfMRI connectivity and dynamics could be spuriously linked to behavioral measures of cognitive functions via arousal-related rsfMRI changes. Although this is purely a conjecture to be tested by future studies, caution needs to be exercised before completely excluding this possibility.

## Concluding Remarks and Future Research

Here we reviewed the current understanding of the relationship between the brain arousal and resting-state fMRI, in particular a newly discovered neurophysiological event underlying the global rsfMRI signal. We then discussed potential implications of the arousal-related modulation on rsfMRI research, i.e., its role in affecting rsfMRI connectivity/dynamics and mediating their spurious correlations with physiological and behavioral measures. We also formulated multiple testable hypotheses based on existing evidence. Future research ought to validate these hypotheses, which are important not only for proper interpretations of rsfMRI results but also for better quantifications of brain connectivity and dynamics using rsfMRI with properly dealing with the arousal confounding effects, i.e., removing or retaining the arousal-related component based on research purposes. Before the validation of these hypothesis, one should be cautious about large global signal and sensory-dominant pattern in rsfMRI results, which are indicative of arousal involvement. Researchers may also consider to reduce the potential arousal influence at the stage of data collection, for example, by acquiring data at the eye-open state or breaking down a long scan into multiple shorter ones. The profound arousal effect on rsfMRI presents additional challenges to rsfMRI-based measures of brain connectivity/dynamics. But on the bright side, this would enable fMRI-based arousal measures (Chang et al., 2016a; Falahpour et al., 2018; Liu et al., 2018), which may provide new opportunities for understanding the arousal’s role in affecting brain function and dysfunction, especially with big neuroimaging data acquired recently from healthy and diseased populations. It is, however, worth noting that the performance of these template-based arousal measures could be dependent on the presence of the SST events and thus the general vigilance state (Falahpour et al., 2018). It remains a challenge for future studies to improve the fMRI-based arousal measure by better understanding arousal-related fMRI changes.

## Author Contributions

YG and XL designed the study and performed the analyses. YG, XL, and FH wrote the manuscript.

## Conflict of Interest

The authors declare that the research was conducted in the absence of any commercial or financial relationships that could be construed as a potential conflict of interest.
